# The Role of Autophagy in Critical Illness-induced Liver Damage

**DOI:** 10.1038/s41598-017-14405-w

**Published:** 2017-10-26

**Authors:** Steven E. Thiessen, Inge Derese, Sarah Derde, Thomas Dufour, Lies Pauwels, Youri Bekhuis, Isabel Pintelon, Wim Martinet, Greet Van den Berghe, Ilse Vanhorebeek

**Affiliations:** 1 Clinical Division and Laboratory of Intensive Care Medicine, Department of Cellular and Molecular Medicine, KU Leuven, Leuven, 3000 Belgium; 2 Laboratory of Cell Biology and Histology, University of Antwerp, Antwerp, 2610 Belgium; 3 Laboratory of Physiopharmacology, University of Antwerp, Antwerp, 2610 Belgium

## Abstract

Mitochondrial dysfunction and endoplasmic reticulum (ER) stress, which activates the unfolded protein response (UPR), mediate critical illness-induced organ failure, often affecting the liver. Autophagy is known to alleviate both and suppressed or insufficiently activated autophagy in prolonged illness has shown to associate with organ failure. Whether insufficient autophagy contributes to organ failure during critical illness by affecting these underlying mechanisms is incompletely understood. In this study, we investigated whether the inability to acutely activate hepatic autophagy during critical illness aggravates liver damage by increasing hepatic mitochondrial dysfunction and affecting the UPR. In a mouse model of critical illness, induced by surgery and sepsis, we investigated the impact of inactivating hepatic autophagy on markers of hepatic mitochondrial function, the UPR and liver damage in acute (1 day) and prolonged (3 days) critical illness. Hepatic autophagy inactivation during critical illness acutely worsened mitochondrial dysfunction and time-dependently modulated the hepatic UPR. Furthermore, autophagy inactivation aggravated markers of liver damage on both time points. In conclusion, the inability to acutely activate autophagy in liver during critical illness worsened hepatic mitochondrial damage and dysfunction, partially prohibited acute UPR activation and aggravated liver damage, indicating that autophagy is crucial in alleviating critical illness-induced organ failure.

## Introduction

Multiple organ failure is a leading cause of morbidity and mortality in critically ill patients, in which the liver is often affected^1^. The pathogenesis of critical illness-induced organ failure and the pathways leading to recovery thereof are incompletely understood. Despite the severe metabolic, hemodynamic and inflammatory stress, organs including the liver, show little signs of cell death during critical illness^2^. Instead, cells of failing organs accumulate damaged and/or dysfunctional organelles, such as mitochondria^3,4^. The cells also accumulate damaged and misfolded proteins, which leads to endoplasmic reticulum (ER) stress^5^. Hepatic ER stress activates the unfolded protein response (UPR), which is an adaptive signaling pathway to minimise the amount of damaged and unfolded proteins. The UPR comprises three branches, the p-eIF2alpha, IRE1alpha-XBP1s and ATF6-CREB3L3 pathways^6–9^. With excessive ER stress the UPR may fail, which promotes cellular dysfunction and cell death^6,7^. Evidence suggests that mitochondrial dysfunction, ER stress and the UPR are potentially important contributors to critical illness-induced organ failure^3–5^.

Autophagy is a process that degrades damaged or abnormal cellular components^10^. This removal is crucial for alleviating mitochondrial damage and ER stress to restore cellular homeostasis, and hence for cell function and survival^11,12^. Interestingly, autophagy inactivation suppresses several branches of the UPR, possibly due to excessive ER stress, hereby promoting cell death^6,7^. Studies on autophagic activity in critical illness have generated at first sight conflicting results. Whereas initial observations were interpreted as increased hepatic autophagy evoked by illness^13,14^, other studies showed insufficiently activated or even suppressed autophagy, the degree of which correlated with mitochondrial dysfunction, liver damage/failure and adverse outcomes^15,16^. Different methods to evaluate autophagy and variable time points studied may have played a role. Indeed, hepatic autophagy might be activated in the acute phase, but suppressed with prolonged critical illness^17^. This may suggest that an immediate up-regulation of hepatic autophagy during critical illness is an adaptive response to clear damaged mitochondria and protein aggregates, and when insufficient, lingering liver damage/failure may persist in the prolonged phase of illness. If so, this would mean that autophagy is a crucial pathway in alleviating critical illness-induced organ failure with therapeutic perspectives.

Therefore, we hypothesised that an immediate activation of hepatic autophagy in response to critical illness is an essential adaptive response to prevent further and persistent mitochondrial and liver dysfunction and damage. To test this hypothesis, we compared critical illness, induced by a combination of surgery and sepsis, in wild-type mice and mice with inducible liver-specific knock-out of autophagy-related gene-7 (*Atg7*), which inactivates autophagy. We evaluated the impact of the inability to activate hepatic autophagy in response to critical illness on markers of mitochondrial dysfunction and damage, the UPR and liver damage in the acute and prolonged phase of critical illness.

## Results

### Autophagy deficiency in liver during critical illness aggravated hepatic mitochondrial dysfunction

**Figure 1:**
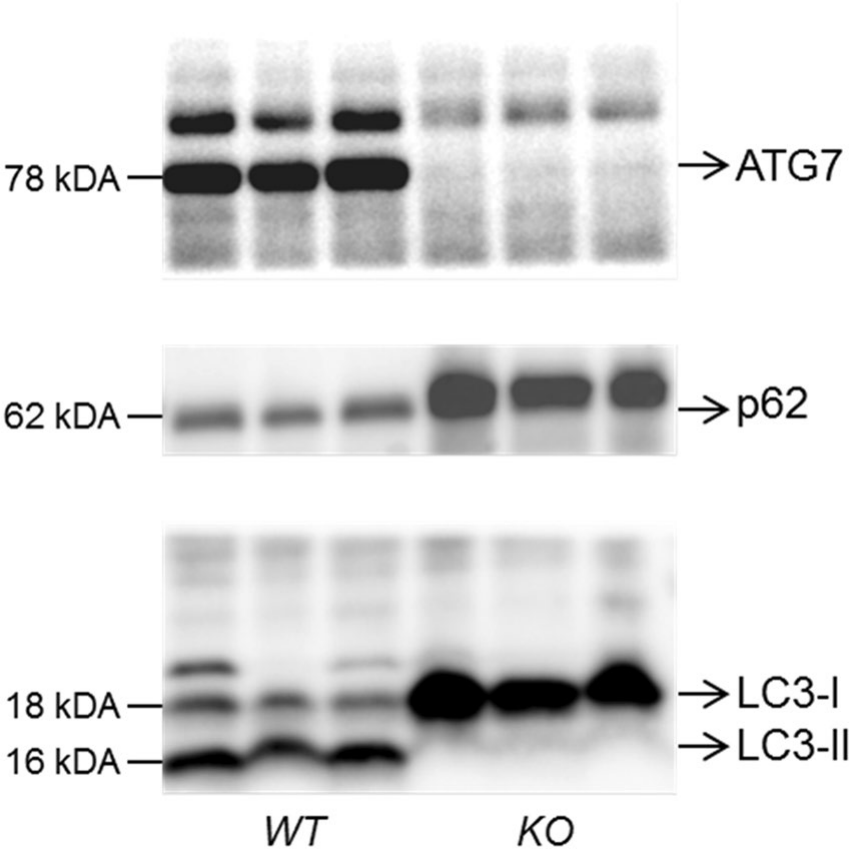
Confirmation of loss of hepatic ATG7 protein and a phenotype of hepatic autophagy inactivation. Fourty µg of liver protein were loaded on 4–20% gradient Tris-Glycine gels and subsequently immunoblotted with antibodies against ATG7, p62 and LC3. Blots were visualised with G:BOX Chemi XRQ (SynGene). Representative immunoblots are shown for WT and KO mice. Full-length immunoblots are presented in Supplementary Figure S1. ATG7: autophagy-related protein 7; p62: sequestosome 1; LC3: microtubule-associated protein 1 light chain 3; WT: *Mx1-Cre* ^−^ *Atg7* ^*F*/*F*^ mouse; KO: *Mx1-Cre* ^+^ *Atg7* ^*F*/*F*^ mouse.

We studied 2 groups of critically ill and 2 groups of “healthy” pair-fed mice for 1 day (“acute phase”) and 3 days (“prolonged phase”). Each time, autophagy was inactivated in one group, but not in the other. Autophagy deficiency was validated by loss of ATG7 protein, impaired LC3-II formation and accumulation of p62 as autophagy substrate (Fig. 1)^18^.

**Figure 2:**
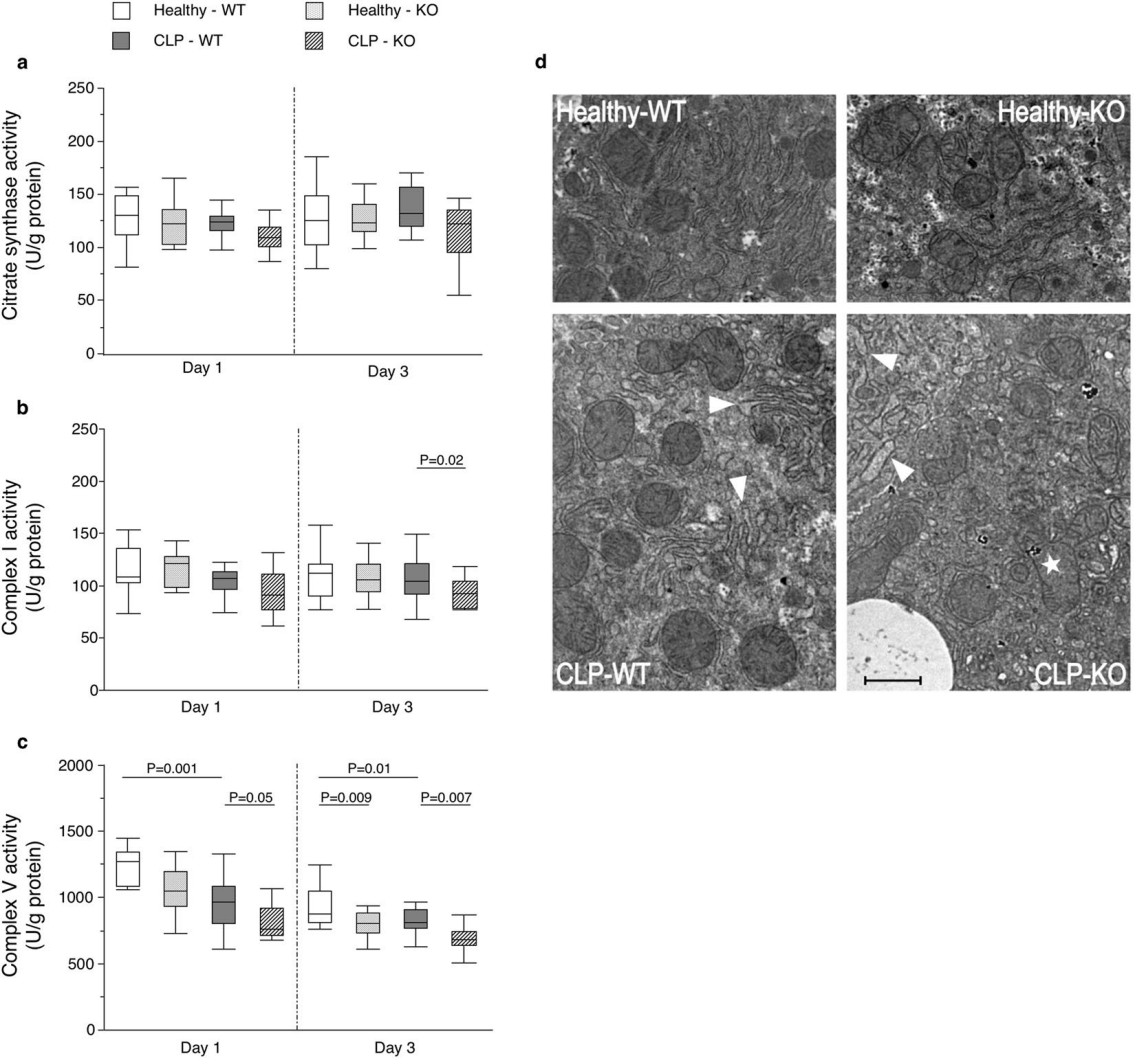
Effect of hepatic autophagy inactivation on hepatic mitochondrial function and morphology in critically ill mice. Hepatic citrate synthase activity (**a**), mitochondrial complex I activity (**b**) and mitochondrial complex V activity (**c**) are shown for healthy pair-fed WT mice (white box plots), healthy pair-fed KO mice (dotted box plots), critically ill WT mice (gray box plots) and critically ill KO mice (hatched box plots) at day 1 and day 3. Box plots depict medians with interquartile ranges (IQR) and whiskers are drawn to the furthest point within 1.5 × IQR from the box. n = 10–16 per group. Representative transmission electron microscopy photographs of the liver (**d**) are shown for healthy pair-fed WT mice (upper left panel), healthy pair-fed KO mice (upper right panel), critically ill WT mice (lower left panel) and critically ill KO mice (lower right panel) at day 3 after induction of critical illness. Arrowheads represent dilated endoplasmic reticulum, whereas the star represents a morphologically abnormal mitochondrion. The scale bar represents 1 µm. KO: *Mx1-Cre* ^+^ *Atg7* ^*F*/*F*^ mouse; WT: *Mx1-Cre* ^−^ *Atg7* ^*F*/*F*^ mouse.

Critical illness did not affect the activity of citrate synthase or of the mitochondrial respiratory chain complex I at the studied time points, whereas complex V activity (responsible for ATP synthesis^19^) was decreased (Fig. 2, Supplementary Table S1). Hepatic autophagy inactivation during critical illness further reduced complex V activity in the acute phase and reduced complex I and complex V activities in the prolonged phase. The pronounced mitochondrial dysfunction in the prolonged phase when functional autophagy was absent coincided with a higher abundance of morphologically abnormal mitochondria (Fig. 2d).

**Figure 3:**
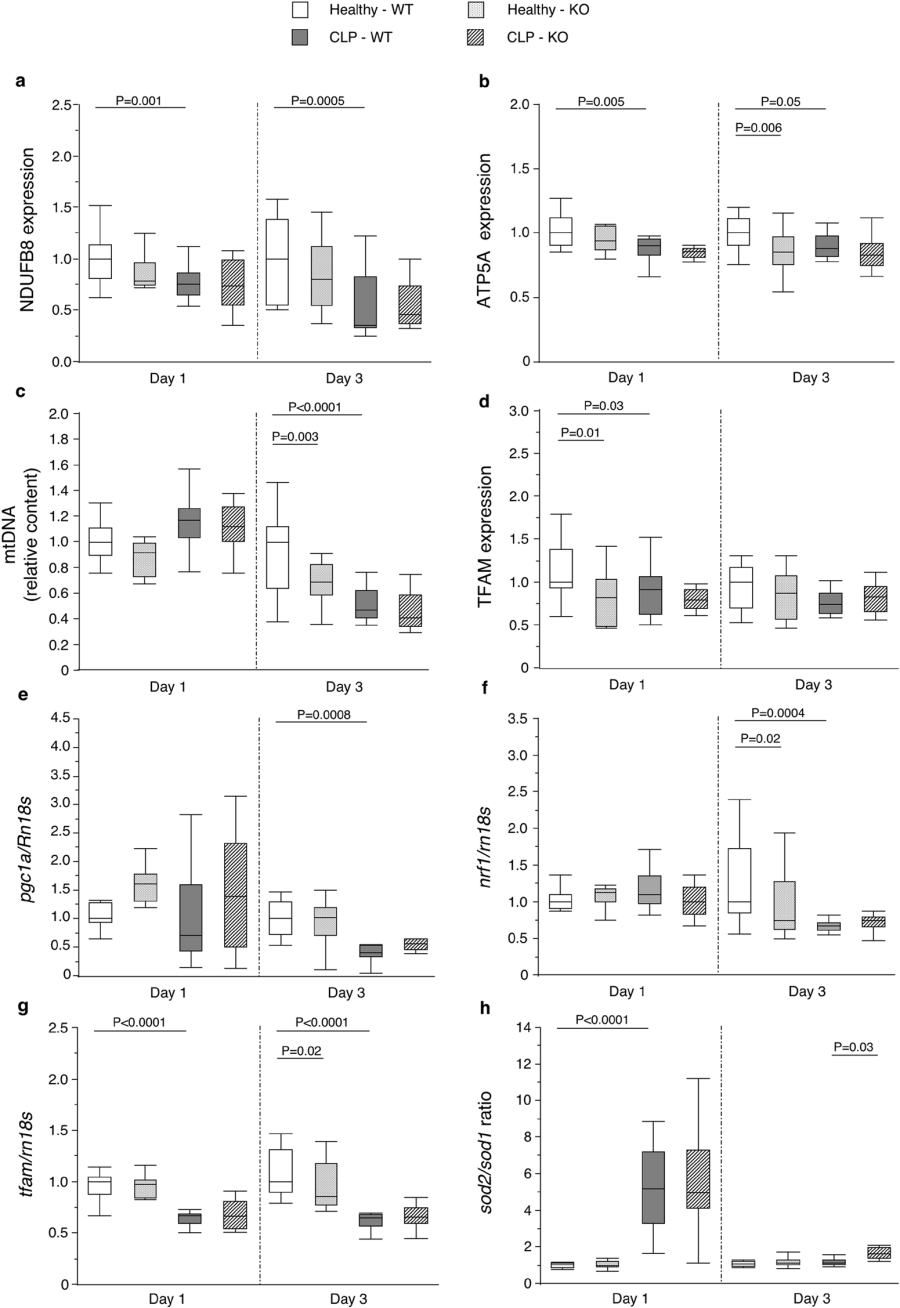
Effect of hepatic autophagy inactivation on markers of hepatic mitochondrial content, mitochondrial biogenesis and mitochondrial oxidative stress in critically ill mice. Hepatic protein expression of NDUFB8, a subunit of mitochondrial complex I (**a**), ATP5A, a subunit of mitochondrial complex V (**b**), hepatic mtDNA content (**c**), hepatic protein expression of TFAM (**d**), hepatic mRNA expression of *pgc1α* (**e**), *nrf1* (**f**), *tfam* (**g**), all markers of mitochondrial biogenesis, and the ratio of *sod2* mRNA expression over *sod1* mRNA expression, a marker of mitochondrial oxidative stress (**h**) are shown for healthy pair-fed WT mice (white box plots), healthy pair-fed KO mice (dotted box plots), critically ill WT mice (gray box plots) and critically ill KO mice (hatched box plots) at day 1 and day 3. Box plots depict medians with interquartile ranges (IQR) and whiskers are drawn to the furthest point within 1.5 × IQR from the box. n = 10–16 per group. NDUFB8: NADH dehydrogenase 1 beta subcomplex subunit 8; ATP5A: ATP synthase subunit alpha; mtDNA: mitochondrial deoxyribonucleic acid; *sod1*: superoxide dismutase-1; *sod2*: superoxide dismutase-2; *pgc1α*: peroxisome proliferator-activated receptor gamma coactivator 1-alpha; *nrf1:* nuclear respiratory factor 1; *tfam*, TFAM*:* mitochondrial transcription factor A; *rn18s*: 18 S ribosomal RNA. KO: *Mx1-Cre* ^+^ *Atg7* ^*F*/*F*^ mouse; WT: *Mx1-Cre* ^−^ *Atg7* ^*F*/*F*^ mouse.

To further investigate whether the marked decrease in mitochondrial function by autophagy inactivation could, at least partly, be explained by an effect on mitochondrial content, we assessed protein expression of subunits of the mitochondrial complexes and relative mtDNA copy number as markers of mitochondrial content, and also investigated several markers of mitochondrial biogenesis (Supplementary Table S1). Critical illness decreased the expression of subunits of complex I and V in both phases (Fig. 3a,b) and decreased mtDNA content in the prolonged phase (Fig. 3c). Expression of mitochondrial transcription factor A (TFAM) as downstream marker of the mitochondrial biogenesis pathway was reduced in the acute phase, followed by reductions in upstream markers in the prolonged phase (Fig. 3d–g). Hepatic autophagy inactivation during critical illness did not significantly affect any of these markers at any time point.

To evaluate whether autophagy deficiency aggravated mitochondrial oxidative stress during critical illness, which may have contributed to the decreased mitochondrial function, gene expression of superoxide dismutase-2 (*sod2*), a key mitochondrial antioxidant enzyme, was quantified and compared with that of the cytoplasmic *sod1*, as previously described^20^. Critical illness increased the *sod2*/*sod1* ratio in the acute, but not in the prolonged phase (Fig. 3h, Supplementary Table S1). Hepatic autophagy inactivation during critical illness increased this ratio in the prolonged phase.

### Autophagy deficiency in liver during critical illness altered the UPR

**Figure 4:**
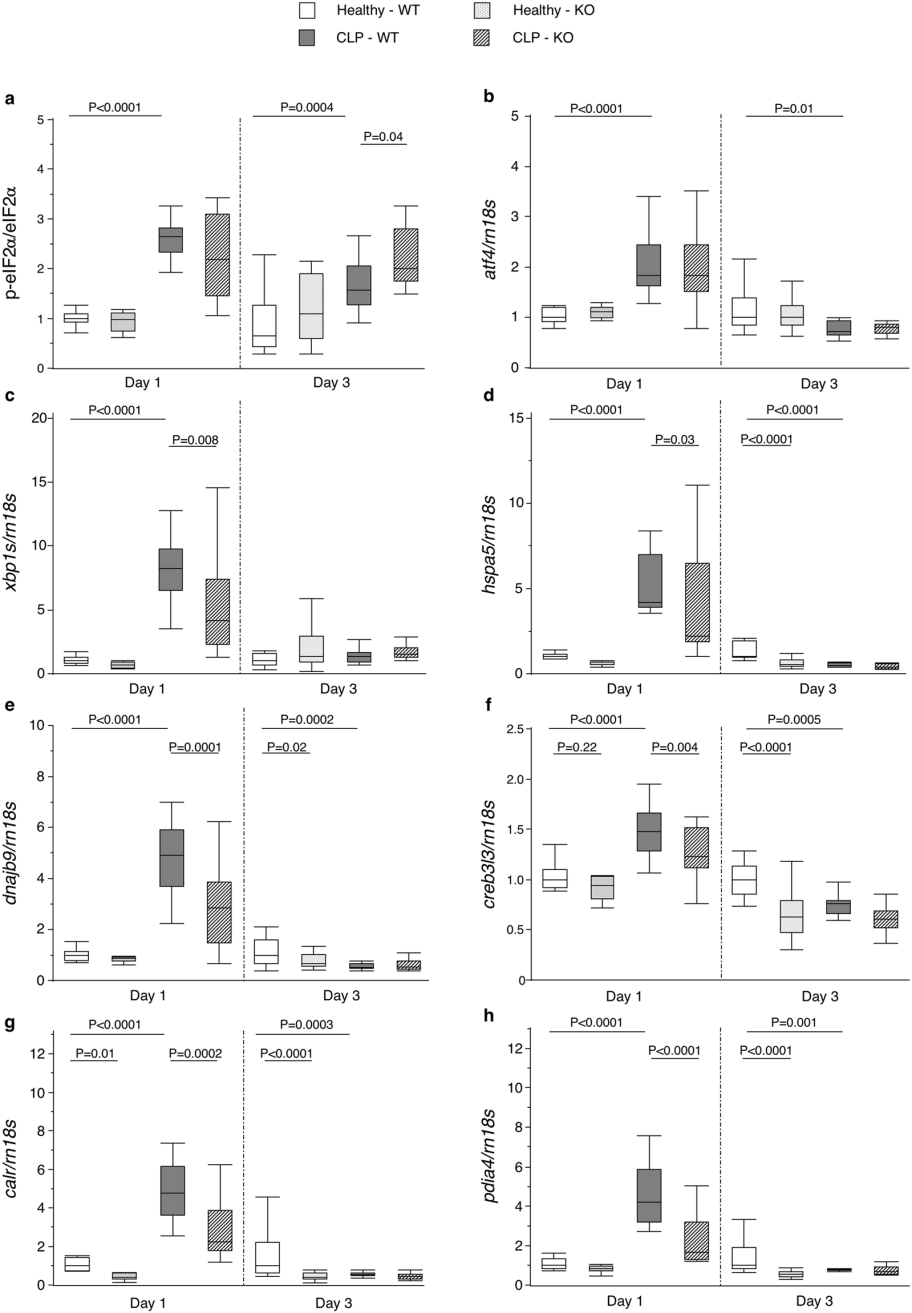
Effect of hepatic autophagy inactivation on markers of the hepatic UPR in critically ill mice. The ratio of hepatic p-eIF2alpha over eIF2alpha protein expression (**a**), hepatic mRNA expression of *atf4* (**b**), *xbp1s* (**c**), *hspa5* (**d**), *dnajb9* (**e**), *creb3l3* (**f**), *calr* (**g**) and *pdia4* (**h**) are shown for healthy pair-fed WT mice (white box plots), healthy pair-fed KO mice (dotted box plots), critically ill WT mice (gray box plots) and critically ill KO mice (hatched box plots) at day 1 and day 3. Box plots depict medians with interquartile ranges (IQR) and whiskers are drawn to the furthest point within 1.5 × IQR from the box. n = 10–16 per group. p-eIF2alpha: phosphorylated eukaryotic initiation factor 2 alpha; eIF2alpha: eukaryotic initiation factor 2 alpha; *atf4*: activating transcription factor 4; *xbp1s*: spliced form of x-box binding protein 1; *hspa5*: heat shock protein family A member 5; *dnajb9*: dnaJ heat shock protein family member B9; *creb3l3*: cAMP responsive element binding protein 3-like 3; *calr*: calreticulin; *pdia4*: protein disulfide isomerase family A member 4. KO: *Mx1-Cre* ^+^ *Atg7* ^*F*/*F*^ mouse; WT: *Mx1-Cre* ^−^ *Atg7* ^*F*/*F*^ mouse.

We evaluated markers of the 3 UPR branches in liver (Supplementary Table S1). Critical illness increased the p-eIF2alpha/eIF2alpha ratio at both time points (Fig. 4a). This was accompanied by a rise in *atf4* expression as downstream target in the acute phase, but by a decrease in the prolonged phase (Fig. 4b). *Xbp1s* expression, a marker of an activated IRE1alpha-XBP1s pathway, was increased during critical illness in the acute, but not in the prolonged phase (Fig. 4c). The expression of *hspa5* and *dnajb9* as chaperones transcriptionally regulated by XBP1s was similarly elevated in the acute phase, but down-regulated in the prolonged phase (Fig. 4d,e). Likewise, the expression of *creb3l3* as marker of the third UPR branch was up-regulated in the acute phase and down-regulated in the prolonged phase of critical illness (Fig. 4f). ATF6-CREB3l3 downstream targets *calr* and *pdia4* followed the same pattern (Fig. 4g,h). Even though several UPR branches appeared not to be up-regulated in the prolonged phase of critical illness, severe ER stress was suggested by the remarkable presence of distended ER (Fig. 2d).

Autophagy deficiency during critical illness time-dependently modulated the UPR. In the acute phase, autophagy deficiency did not affect the p-eIF2alpha/eIF2alpha ratio or *atf4* expression, but decreased the expression of *xbp1s* and *creb3l3* and their downstream targets (Fig. 4). In the prolonged phase, autophagy deficiency increased the p-eIF2alpha/eIF2alpha ratio, but did not affect the markers of the other UPR branches (Fig. 4). These mice also showed distended ER as the critically ill wild-type mice (Fig. 2d).

**Figure 5:**
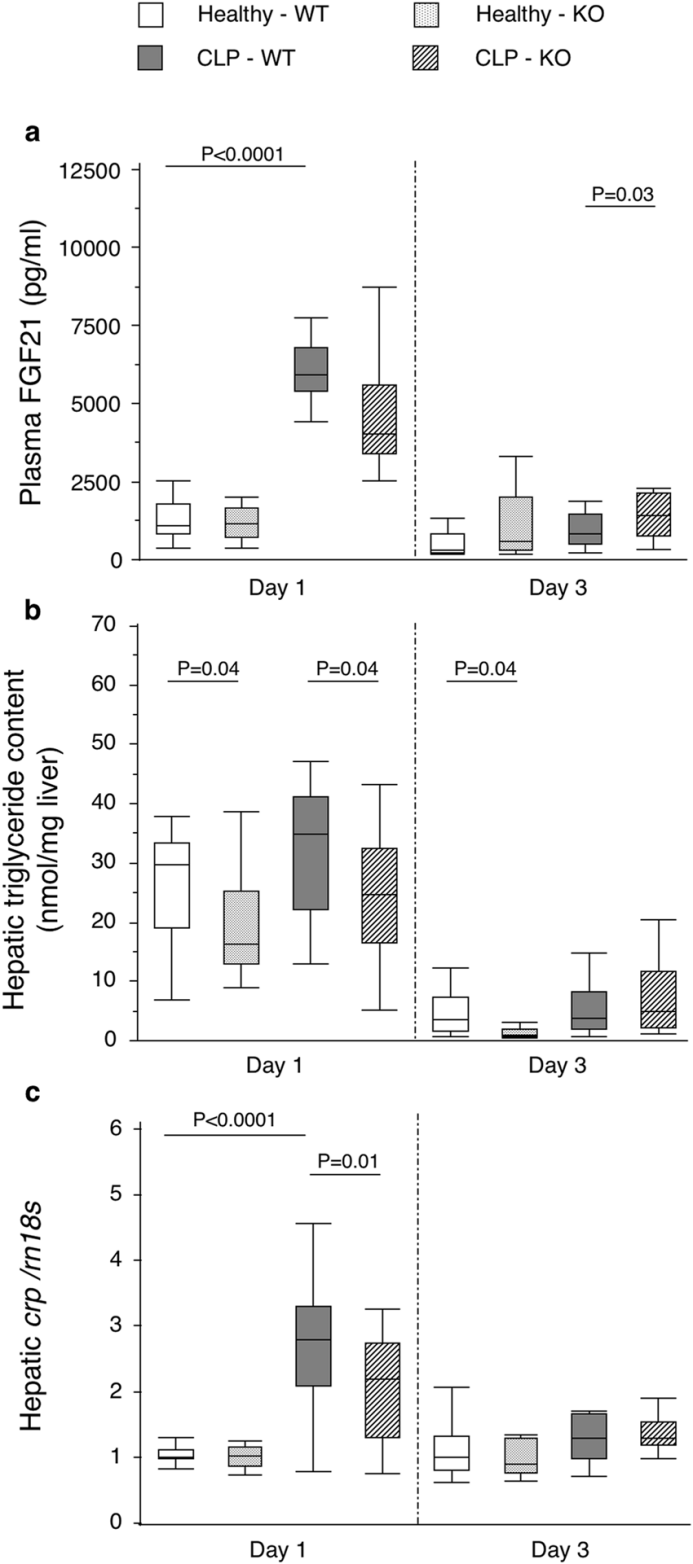
Effect of hepatic autophagy inactivation on plasma FGF21, hepatic triglyceride content and *crp* expression in critically ill mice. Plasma concentration of FGF21 (**a**), hepatic triglyceride content (**b**) and hepatic mRNA expression of *crp* (**c**) are shown for healthy pair-fed WT mice (white box plots), healthy pair-fed KO mice (dotted box plots), critically ill WT mice (gray box plots) and critically ill KO mice (hatched box plots) at day 1 and day 3 after induction of critical illness. Box plots depict medians with interquartile ranges (IQR) and whiskers are drawn to the furthest point within 1.5 × IQR from the box. n = 10–16 per group. FGF21: fibroblast-growth-factor-21; *crp*: C-reactive protein. KO: *Mx1-Cre* ^+^ *Atg7* ^*F*/*F*^ mouse; WT: *Mx1-Cre* ^−^ *Atg7* ^*F*/*F*^ mouse.

As the UPR regulates the systemic inflammatory response and metabolism^7,9,21^, we evaluated several related markers that are affected by critical illness and by the UPR in relation to the time-dependent UPR modulation by autophagy deficiency. These included serum concentrations of FGF21, a recently identified hormone regulating metabolism, hepatic triglyceride content and C-reactive protein (*crp*) gene expression (Supplementary Table S1). Critical illness highly increased plasma FGF21 concentrations in the acute, but not in the prolonged phase (Fig. 5a). Hepatic triglyceride content was not significantly affected by critical illness in this model (Fig. 5b). Hepatic *crp* expression was increased in the acute, but not in the prolonged phase (Fig. 5c). Autophagy deficiency during critical illness decreased hepatic triglyceride content and *crp* expression in the acute phase and increased plasma FGF21 concentrations in the prolonged phase (Fig. 5a–c).

### Autophagy deficiency in liver during critical illness aggravated liver damage and apoptosis

**Figure 6:**
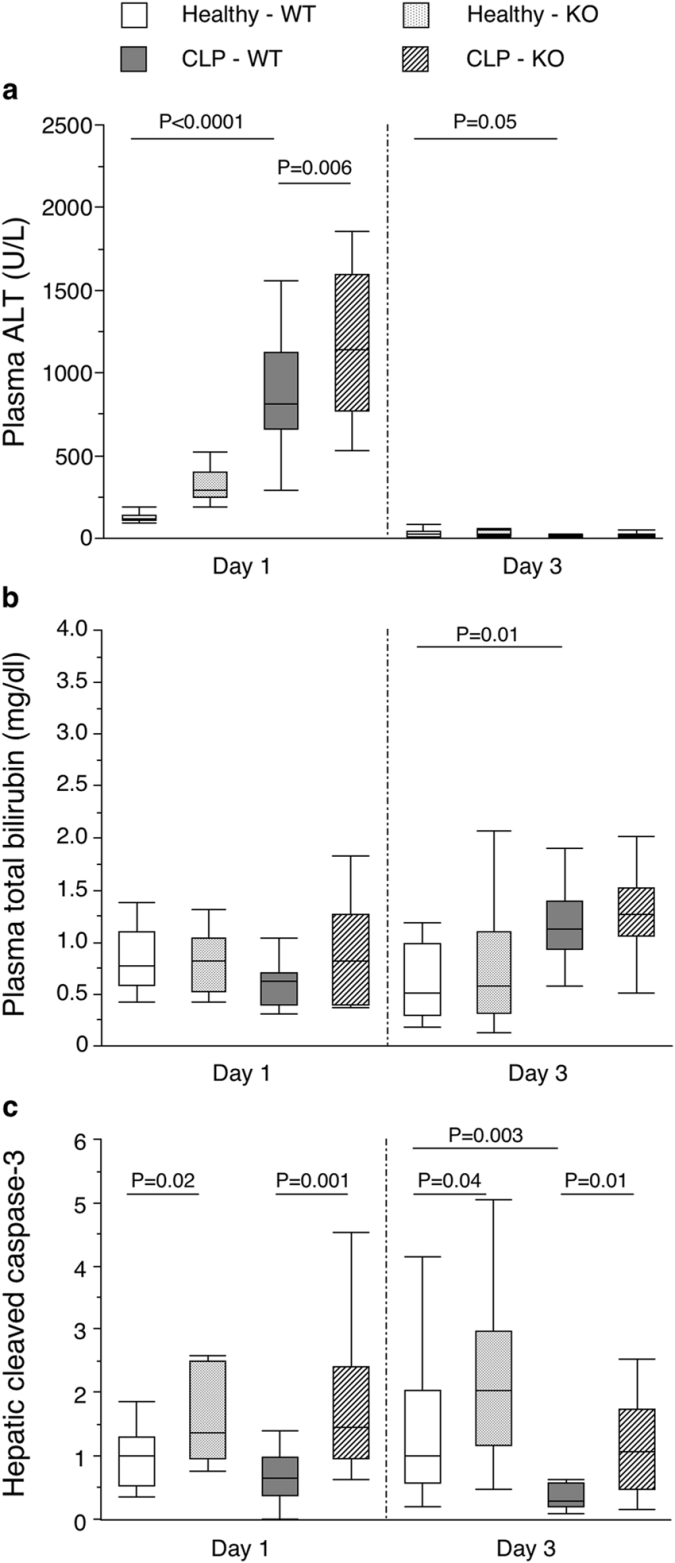
Effect of hepatic autophagy inactivation on markers of hepatic damage in critically ill mice. Plasma concentrations of ALT (**a**) and bilirubin (**b**) and hepatic protein expression of cleaved caspase-3 (**c**) are shown for healthy pair-fed WT mice (white box plots), healthy pair-fed KO mice (dotted box plots), critically ill WT mice (gray box plots) and critically ill KO mice (hatched box plots) at day 1 and day 3. n = 10–16 per group. Box plots depict medians with interquartile ranges (IQR) and whiskers are drawn to the furthest point within 1.5 × IQR from the box. ALT: alanine-aminotransferase. KO: *Mx1-Cre* ^+^ *Atg7* ^*F*/*F*^ mouse; WT: *Mx1-Cre* ^−^ *Atg7* ^*F*/*F*^ mouse.

Critical illness increased plasma concentrations of ALT as marker of hepatic injury in the acute, but no longer in the prolonged phase (Fig. 6a, Supplementary Table S1). Plasma bilirubin as marker of cholestasis was elevated in the prolonged phase (Fig. 6b). Hepatic autophagy inactivation during critical illness further increased plasma ALT in the acute phase (Fig. 6a) without an effect on bilirubin (Fig. 6b). Critical illness did not increase hepatic expression of the apoptosis marker cleaved caspase-3 (Fig. 6c). However, hepatic autophagy inactivation during critical illness increased cleaved caspase-3 expression at both time points. Survival after 1 and 3 days was unaffected (Table 1).

**Table 1:** Survival status.

Group	Day 1		Day 3	
	Included	Survived	Included	Survived
H-WT	15	15	15	15
H-KO	15	15	15	15
CLP-WT	16	16	17	15
CLP-KO	17	16	15	15

H: healthy; CLP: caecal ligation and puncture; KO: *Mx1-Cre* ^+^ *Atg7* ^*F*/*F*^ mouse; WT: *Mx1-Cre* ^−^ *Atg7* ^*F*/*F*^ mouse.

## Discussion

Inability to activate hepatic autophagy in response to critical illness induced by a combination of surgery and sepsis in mice further aggravated functional and ultrastructural mitochondrial damage accompanied by more pronounced mitochondrial oxidative stress, partially suppressed the initial UPR activation with metabolic and inflammatory consequences, and further increased liver injury markers.

Autophagy is an important cellular defense mechanism indispensable for maintaining cellular homeostasis in stress situations^10^. The current study suggests that autophagy activation is also important in critical illness. Indeed, in the acute phase of critical illness increased signs of hepatic damage and apoptosis were present when autophagy could not be activated, suggesting a hepatoprotective adaptive response. Such an acute hepatoprotective effect is in agreement with earlier findings that identified heme oxygenase-1-mediated autophagy as protective against hepatic damage and apoptosis in the early phase during sepsis^22^. Our study is the first to show that autophagy remains hepatoprotective in the prolonged phase of critical illness.

Underlying mechanisms of autophagy-induced hepatoprotection in sepsis had not been thoroughly investigated. Several mechanisms may contribute to the detrimental effect of autophagy inactivation on hepatocyte integrity and survival. Mitochondrial dysfunction is currently considered an important contributor to critical illness-induced organ damage/failure and may compromise cellular energy provision and increase oxidative stress^23^. Autophagy deficiency during critical illness could hamper removal of such damaged/dysfunctional mitochondria. Indeed, in otherwise healthy mice hepatic genetic autophagy inactivation resulted in the accumulation of deformed mitochondria and impaired mitochondrial function^21^. We observed such aggravation of mitochondrial dysfunction when hepatic autophagy was genetically inactivated during critical illness. This was more pronounced in the prolonged phase of illness, where also a higher abundance of deformed mitochondria was observed, associated with signs of persistent mitochondrial oxidative stress, which may have contributed to the mitochondrial dysfunction. Interestingly, the aggravated mitochondrial dysfunction was not explained by an effect on surrogate markers of mitochondrial content, as expression of mitochondrial complex subunits and the mtDNA content were unaltered. Since mitochondrial content is the net result of removal and biogenesis of mitochondria, and as hepatic mitochondrial biogenesis has shown to depend on autophagy during sepsis, we also evaluated markers of mitochondrial biogenesis^24^. However, hepatic autophagy inactivation during critical illness did not affect mitochondrial biogenesis at the investigated time points. These data suggest that autophagy deficiency during critical illness suppressed mitochondrial function by not eliminating the critical illness-induced damaged mitochondria via mitophagy.

Another potential explanation for the aggravation of hepatic damage and apoptosis by autophagy deficiency may lie in its effect on ER stress and the UPR modulation. When ER is damaged and/or abnormal protein aggregates accumulate in the ER, ER stress activates the UPR, in turn activating autophagy to eliminate the damaged ER and protein aggregates^12,25^. Hence, when autophagy cannot be activated as in this *Atg7* knock-out model, this could lead to more severe ER stress^20,26^. Prolonged or excessive ER stress, in turn, can suppress the adaptive UPR response, resulting in cell death^6^. Indeed, severe ER stress can attenuate the IRE1alpha-XBP1s and ATF6-CREB3L3 pathways, together with a sustained p-eIF2alpha pathway, which has been shown to induce apoptosis^6^. In our study, markers of the three branches of the UPR were increased in the acute phase of critical illness. However, in the prolonged phase and despite clear ultrastructural signs of ER stress, the up-regulation of the IRE1alpha-XBP1s and the ATF6-CREB3L3 pathway was lost while the p-eIF2alpha pathway remained activated. These findings could point to an exhaustion of the adaptive UPR in the prolonged phase of critical illness, possibly due to excessive ER stress, and suggest that inadequate UPR activation may play an important role in mediating organ damage/failure during critical illness. We found a similar pattern of UPR modulation by hepatic autophagy deficiency in the acute phase of critical illness in the presence of signs of increased apoptosis and markers of hepatic injury. Indeed, the critical illness-induced rise in the IRE1alpha-XBP1s and ATF6-CREB3L3 pathways was attenuated in the *Atg7*-deficient ill mice, whereas increased activation of the p-eIF2alpha pathway was preserved. Thus, these findings could point to an exhaustion of the adaptive UPR when hepatic autophagy cannot be activated during critical illness, which can lead to hepatocyte death. In prolonged critical illness, autophagy deficiency did not affect the IRE1alpha-XBP1s and ATF6-CREB3L3 pathways, while stimulating the p-eIF2alpha in this phase, although not up to the level of atf4 expression. Interestingly, mitochondrial dysfunction can also stimulate the p-eIF2alpha pathway^21^. Therefore, the increased hepatic p-eIF2alpha expression by autophagy inactivation in prolonged critical illness could also result from the markedly aggravated mitochondrial dysfunction in these mice.

Interestingly, previous reports have shown that autophagy mediates cellular resistance to lipopolysaccharide-induced tumor necrosis factor toxicity, by blocking hepatic apoptotic pathways^27,28^. A detrimental effect of autophagy inactivation on mitochondrial function and ER homeostasis, as shown here, might have played a role.

Besides determining cell faith, the UPR can affect metabolic and inflammatory responses^7,9^. In that regard, attenuation of the ATF6-CREB3L3 pathway has shown to prevent hepatic triglyceride accumulation during fasting and to decrease the inflammatory response^7,9^. As for the UPR, we also found time-dependent alterations in metabolic and inflammatory markers with deficient autophagy during critical illness, which may be related to the UPR modulation. In the acute phase of illness, autophagy deficiency lowered hepatic triglyceride content and crp expression, concomitant with the previously discussed decrease in markers of the ATF6-CREB3L3 pathway. This suggests that the ATF6-CREB3L3 pathway may be involved in some of the metabolic and inflammatory responses to critical illness. Activation of the p-eIF2alpha pathway, which can also be triggered by mitochondrial dysfunction^21^, coincided with higher plasma concentrations of FGF21, a recently identified stress hormone. This may suggest that FGF21 is in part increased via this pathway, possibly as an adaptive response to mitochondrial dysfunction, which is in accordance with previous findings^23,29^. Further research is warranted to identify the role of ER stress and the UPR in the metabolic and inflammatory response during critical illness.

As in previous studies with otherwise healthy autophagy-deficient mice^18^, we also observed a pronounced increase in p62 protein content when autophagy was inactivated during critical illness. Accumulation of p62 can evoke several disturbances in liver, such as chronic inflammation, metabolic reprogramming, and tumourigenesis^30,31^. Hence, at least part of the effects of hepatic autophagy deficiency in our mouse model of critical illness, such as the increased liver damage and altered metabolic and inflammatory responses, may be indirectly explained by the pronounced p62 accumulation. Also in liver biopsies of critically ill human patients, such a pronounced accumulation of p62 has been observed together with signs of liver damage^16^. Further research is therefore warranted to identify the role of p62 during critical illness.

Our findings have important clinical implications. By identifying autophagy as a key player in regulating organ damage/function and the metabolic and inflammatory response to critical illness induced by a combination of surgery and sepsis, these findings open new therapeutic perspectives. On one hand, identifying interventions that could stimulate autophagy in critically ill patients may improve the outcome of these patients. Unfortunately, no clinically useful autophagy activators are available for administration to critically ill patients at this moment. Further research is therefore warranted to identify such agents. On the other hand, this insight supports the concept of avoiding inhibition of autophagy to improve the outcome of critically ill patients. Interestingly, early administration of (parenteral) nutrition, as powerful physiological suppressor of autophagy, has shown to aggravate organ dysfunction in critical illness, to attenuate the rise in plasma CRP and to slow down recovery from critical illness^32–34^. Our data might offer a mechanistic explanation for these findings, implying suppression of autophagy, and support the notion to not start nutrition early during critical illness.

Our study has some limitations to highlight. First, although the mouse model mimics several characteristics of human critical illness, one should always be cautious to extrapolate experimental findings to the human setting. We used the CLP procedure as golden standard to induce sepsis, which actually requires surgery to induce sepsis. As surgery and sepsis often co-occur in critically ill patients, we aimed to study the impact of critical illness rather than the separate impact of the sepsis component. Hence, we did not include a surgical control group. Second, we investigated markers of the UPR as an adaptive response to ER stress. However, since techniques to reliably evaluate ER stress *in vivo* are lacking, we could not directly assess the effect of autophagy deficiency on ER stress. Third, to better mimic the human situation, the critically ill mice received parenteral nutrition in the prolonged phase of critical illness. Although nutrition is known to affect autophagy during critical illness^33,34^, its administration did not confound our present findings, since both the autophagy-deficient and wild-type mice were equally fed.

In conclusion, the inability to acutely activate autophagy in liver during critical illness in mice worsened hepatic mitochondrial damage and dysfunction, partially prohibited acute UPR activation and aggravated liver damage. Autophagy deficiency also affected some metabolic and inflammatory responses to critical illness, possibly mediated via its effect on the UPR. These findings suggest that an acute activation of hepatic autophagy in response to critical illness is an adaptive response to prevent liver damage/failure, possibly by preventing accumulating damaged mitochondria and hereby mitochondrial dysfunction and by modulating the UPR. These findings provide support for further investigating the potential of autophagy activators for attenuating critical illness-induced organ failure and to enhance recovery.

## Methods

### Animals

We used 24-weeks old mice with inducible inactivation of *Atg7* in liver (*Mx1-Cre* ^+^ *Atg7* ^*F*/*F*^ mice) in which hepatic autophagy is lost upon treatment with polyinosinic acid–polycytidylic acid (pIpC, Sigma), referred to as liver-specific autophagy-deficient (KO) mice, and *Mx1-Cre* ^−^ *Atg7* ^*F*/*F*^ mice, referred to as wild-type (WT) mice^18^. These mice were bred starting from two mouse strains, the *Atg7* ^*F*/*F*^ mouse strain (Riken) and the *Mx1-Cre* mouse strain (Jackson)^18^. Tail genotyping was performed as described^18^. All mice received 5 pIpC doses (450 µl i.p., 1 mg/ml) at 48h-intervals, starting 10 days before inclusion.

We studied 2 groups of critically ill and 2 groups of “healthy” pair-fed mice for 1 day (“acute phase”) and 3 days (“prolonged phase”). Each time, autophagy was inactivated in one group, but not in the other. Mice were randomly allocated to “healthy” control or critical illness per genotype. Mice in the critically ill groups were subjected to surgery and single-puncture caecal ligation and puncture (CLP) that induced sepsis^35^. Briefly, these mice were anesthetised, a catheter was inserted in the central jugular vein and the surgical CLP procedure was performed (50% ligation of the cecum at half the distance between the distal pole and the base of the cecum and a single puncture through-and-through), which was followed by intravenous fluid resuscitation^36^. Ill mice of the day 3 group received parenteral nutrition from the morning after surgery (5.8 kcal/day, Oliclinomel, Baxter) to mimic the human clinical situation, keeping in mind the higher metabolic rate of mice as compared to humans^33,37^. Ill mice received pain medication and antibiotics throughout the experiment. At sacrifice, blood was withdrawn (cardiac puncture), followed by decapitation and removal of organs, which were snap-frozen in liquid nitrogen and stored at −80 °C. After inclusion of at least 15 surviving animals per group (Table 1), genotyping was repeated on liver and autophagy deficiency was validated by loss of ATG7 protein, impaired LC3-II formation and accumulation of p62 as autophagy substrate (Fig. 1)^18^. Only mice with correct genotype and autophagy status were analyzed.

The KU Leuven Animals Ethics Committee approved the study (P076/2012). Animals were treated according to the “Guide for the Care and Use of Laboratory Animals” prepared by the US National Institutes of Health.

### Analyses on plasma and liver

Plasma concentrations of alanine-aminotransferase (ALT) (Abcam, ab105137), and bilirubin (Diazyme, DZ150A-K) and hepatic triglyceride content (Abcam, ab65336) were measured with colorimetric assays and plasma fibroblast-growth-factor-21 (FGF21) with ELISA (R&D Systems, MF2100).

Citrate synthase and mitochondrial respiratory chain complex activities were measured with spectrophotometry, with enzymatic reactions carried out at 30 °C and followed by the change in absorbance detected with a Lambda 25 UV/VIS spectrometer (Perkin Elmer, Norwalk, CT, USA)^4,38,39^. Liver homogenates (1:15 w/v) were prepared from 50–70 mg tissue in homogenisation buffer [210 mmol/l mannitol, 70 mmol/l sucrose, 5 mmol/l hydroxyethyl piperazineethanesulfonic acid (HEPES), 1 mmol/l ethylene glycol bis(2-aminoethylether) tetra-acetic acid (EGTA), pH 7.2] with use of a borosilicate glass hand homogeniser in ice and subjected to five freeze-thaw cycles. For measurement of citrate synthase and complex I activity, an additional sonication step was performed while on ice (4 cycles of 10 s sonication separated by a 20 s break, Vibra Cell^TM^ VC250, Sonics & Materials Inc, Newtown, CT). For calculation of the activities results for blank reactions were subtracted from the total change in absorbance. Citrate synthase activity was assayed in a buffer containing 50 mM potassium phosphate pH 7.4 and 100 µmol/l 5,5′-dithio-bis(2-nitrobenzoic acid). The reaction was started with addition of sample, 100 µmol/l acetyl-CoA and 100 µmol/l oxaloacetic acid pH 7.2. Oxaloacetic acid was left out for the blank reaction. Production of the thionitrobenzoate anion was followed at 412 nm. Complex I activity was measured in a buffer containing 50 mM potassium phosphate pH 7.4, 50 µmol/l NADH, 1 mmol/l KCN, 10 µmol/l antimycin A, 1 mg/ml bovine serum albumin and 50 µmol/l coenzyme Q_1_. The reaction was started with addition of sample. The complex I inhibitor rotenone (2.5 µmol/l) was added to the blank reaction. The consumption of NADH was followed at 340 nm. For the quantification of complex V activity, we used a 40 mmol/l Tris-bicarbonate buffer with 10 mmol/l EGTA, pH 8.0, to which we added 200 µmol/l NADH, 2.5 mmol/l phosphoenolpyruvate, 5 µmol/l antimycin A, 5 mmol/l magnesium chloride, 27.5 U/ml lactate dehydrogenase, and 10 U/ml pyruvate kinase. After preincubation for 2 min with 2.5 mmol/l ATP, the reaction was initiated by addition of the sample. For the blank reaction 2 µmol/l of the complex V inhibitor oligomycin was added. The consumption of NADH was followed at 340 nm.

Protein isolation and immunoblotting was performed with primary antibodies against ATG7 (Cell Signaling, #2631) eIF2alpha (Cell Signaling, #5324) and cleaved caspase-3 (Cell Signaling; #9664), phosphorylated-eIF2alpha (p-eIF2α, Abcam, ab32157) and mitochondrial respiratory chain complex subunits (Abcam; ab110413), LC3 (Sigma, L7543) and p62 (Novus Biologicals, H00008878-M01)^40^. Secondary horseradish peroxidase-conjugated antibodies were purchased from DakoCytomation. Blots were visualised (G:BOX Chemi XRQ, SynGene) and analyzed with SynGene software. Data were expressed relative to the median of the controls.

mtDNA content was determined on ribonuclease-treated DNA, by amplifying mtDNA-encoded cytochrome c oxidase subunit (*cox2*) and nuclear DNA-encoded amyloid precursor protein (*app*) fragments (Applied Biosystems) (Table 2)^41^.

**Table 2:** Primers and probes for real-time polymerase chain reaction analysis.

Gene Symbol	TaqMan^®^AssayID (Applied biosystems)	
app	Mm03932182_s1	
atf4	Mm00515325_g1	
calr	Mm00482936_m1	
cox2	Mm03294838_g1	
creb3l3	Mm00520279_m1	
crp	Mm00432680_g1	
dnajb9	Mm01622956_s1	
hspa5	Mm00517690_g1	
nrf1	Mm01135606_m1	
pdia4	Mm00437958_m1	
pgc1a	Mm01208835_m1	
rn18s	Mm03928990_g1	
sod1	Mm01344233_g1	
sod2	Mm01313000_m1	
tfam	Mm00447485_m1	
**Gene Symbol**	**Sequence (Eurogentec)**	**Concentration (nM)**
xbp1s	Forward primer: 5′-ctgagtccgcagcaggt-3′	900
	Probe: 5′- ggcccagttgtcacctcccc-3′	300
	Reverse primer: 5′- tgtcagagtccatgggaaga-3′	900

RNA isolation, cDNA synthesis and real-time PCR for gene expression analysis were performed with primers and probes from Applied Biosystems or Eurogentec (Seraing, Belgium) (Table 2)^42^. Relative gene expression was determined (2−ΔΔCt method) with 18 S ribosomal RNA (*rn18S*) as housekeeping gene. Electron microscopy was performed as described^16,43^.

### Statistical analysis

Data are presented as medians and interquartile ranges. Analyses were performed with JMP 12.0 (SAS Institute Inc.). Normally distributed data were compared with ANOVA and if significant followed by each pair Student t-test. Non-normally distributed data were transformed to achieve a near-normal distribution. Two-sided P-values ≤ 0.05 were considered statistically significant.

### Data availability statement

The datasets generated during and/or analysed during the current study are available from the corresponding author on reasonable request.

## Electronic supplementary material

Supplementary information
