# Supplementary material for: The Role of Autophagy in Critical Illness-induced Liver Damage
Source: Sci Rep. 2017 Oct 26;7:14150. doi: 10.1038/s41598-017-14405-w (PMC5658339; doi:10.1038/s41598-017-14405-w)
Supplement: Supplementary file 1 — Supplementary information [file 41598_2017_14405_MOESM1_ESM.pdf]

The Role of Autophagy in Critical Illness-induced Liver Damage

Steven E Thiessen, Inge Derese, Sarah Derde, Thomas Dufour, Lies Pauwels, Youri Bekhuis, Isabel Pintelon, Wim Martinet, Greet Van den Berghe, and Ilse Vanhorebeek

SUPPLEMENTARY INFORMATION

Supplementary Figure S1: Confirmation of loss of hepatic ATG7 protein and a phenotype of hepatic autophagy inactivation

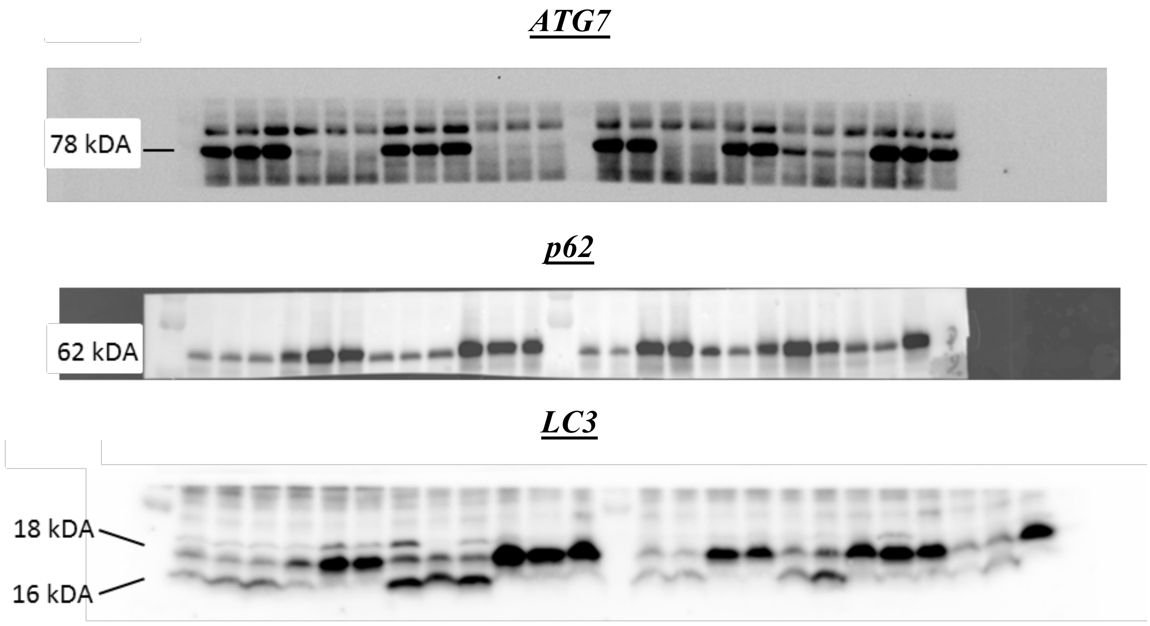

Fourty µg of liver protein were loaded on 4-20% gradient Tris-Glycine gels and subsequently immunoblotted with antibodies against ATG7, p62 and LC3. Blots were visualised with G:BOX Chemi XRQ (SynGene). Full-length immunoblots are shown. The samples derived from the same experiment and gels/blots were processed in parallel. ATG7: autophagy-related protein 7; p62: sequestosome 1; LC3: microtubule-associated protein 1 light chain 3.

Supplementary Table S1: Expansion of abbreviations, description of the function of mentioned markers and effect of critical illness and of autophagy during critical illness on the markers quantified in this study

Abbreviation	Full name	Description of function	Effect of critical illness day 1/day 3	Effect of autophagy deficiency during critical illness day 1/day 3
ALT	alanine aminotransferase	Plasma marker of liver damage	↑ / (↓)	↑ / =
APP [a]	amyloid precursor protein	Nuclear DNA-encoded membrane protein, used in the assay for quantifying mitochondrial DNA content (for normalisation of mtDNA content to nDNA content)		
ATF4 [b]	activating transcription factor 4	Transcription factor, which plays a role in cellular stress defense mechanisms; phosphorylation of eIF2α (vide infra) promotes its translation	↑ / ↓	= / =
ATF6	activating transcription factor 6	Endoplasmic reticulum stress-regulated transmembrane transcription factor, which plays a role in cellular defense mechanisms and affects metabolism and inflammation		
ATG	autophagy-related gene	Genes that encode proteins vital for the proper functioning of autophagy		
ATP5A [c]	ATP synthase subunit alpha	Subunit of the mitochondrial ATP synthase, the enzyme complex responsible for the generation of ATP in the oxidative phosphorylation process	↓ / ↓	= / =
CALR [b]	calreticulin	Multifunctional protein that functions as a chaperone and can bind calcium ions;	↑ / ↓	↓ / =

		its expression is up-regulated by the ATF6-CREB3L3 pathway of the unfolded protein response		
Citrate synthase [d]		Mitochondrial matrix enzyme involved in the Krebs cycle	= / =	= / =
Cleaved CASP3 [c]	caspase-3	Protein that plays a crucial role in apoptosis; its cleaved form being a marker of apoptosis	= / ↓	↑ / ↑
CLP	caecal ligation and puncture	Golden standard method of inducing polymicrobial sepsis in animal models		
Complex I [d]	NADH:ubiquinone oxidoreductase	Enzyme complex of the mitochondrial respiratory chain	= / =	= / ↓
Complex V [d]	ATP synthase	Enzyme complex responsible for mitochondrial ATP production in the oxidative phosphorylation process	↓ / ↓	↓ / ↓
COX2 [a]	cytochrome c oxidase, subunit 2	Mitochondrial DNA-encoded protein involved in the mitochondrial respiratory chain, used in the assay for quantifying mitochondrial DNA content		
CREB3L3 [b]	cAMP-responsive element-binding protein 3-like protein 3	Transcription factor that is cleaved upon endoplasmic reticulum stress and activates acute phase response genes, such as CRP	↑ / ↓	↓ / =
CRP [b]	c-reactive protein	Acute phase protein produced by the liver, used as a marker of inflammation	↑ / =	↓ / =
DNAJB9 [b]	dnaJ heat shock protein family member B9	Protein involved in the endoplasmic reticulum-associated degradation of misfolded proteins and acting as a chaperone; its expression is up-regulated by the IRE1alpha-XBP1s (vide infra) pathway of the unfolded protein response	↑ / ↓	↓ / =

p-eIF2α/ eIF2α [c]	eukaryotic translation initiation factor 2α, ratio of the phosphorylated form over total protein expression	Eukaryotic initiation factor required for the initiation of translation; the phosphorylation of this protein inhibits global protein translation while stimulating the translation of several stress proteins	↑ / ↑	= / ↑
ER	endoplasmic reticulum	Cellular organelle that forms an interconnected network of flatted structures known as cisternae		
FGF21 [c]	fibroblast growth factor 21	A recently discovered, mostly liver-derived hormone that acts as an important metabolic modulator	↑ / =	= / ↑
HSPA5 [b]	heat shock protein family A member 5	Protein involved in the correct folding of proteins and degradation of misfolded proteins; its expression is up-regulated by the IRE1alpha-XBP1s pathway of the unfolded protein response	↑ / ↓	↓ / =
IRE1alpha	serine/threonine-protein kinase/endoribonuclease inositol-requiring enzyme 1 alpha	Enzyme located in the endoplasmic reticulum, which is activated upon ER stress; its activation leads to an alteration of gene expression by splicing of the mRNA encoding the transcription factor XBP1		
LC3 [c]	microtubule-associated protein light chain-3	Protein involved in the elongation step of the autophagy process; the immature form LC3-I is converted by lipidation to the mature form LC3-II, which supports the elongation process; this conversion is blocked in the autophagy-deficient mice that lack ATG7, which is crucial in this conversion		

mtDNA	mitochondrial DNA	mitochondrial DNA	= / ↓	= / =
NDUFB8 [c]	NADH dehydrogenase 1 beta subcomplex subunit 8	Subunit of complex I of the mitochondrial respiratory chain	↓ / ↓	= / =
NRF1	nuclear respiratory factor 1	Transcription factor that is a key activator of mitochondrial biogenesis	= / ↓	= / =
p62 [c]	sequestosome-1	Protein with a ubiquitin- and LC3-binding site, that serves as a receptor to deliver cargo for degradation to the autophagosome; it also is involved in several other processes such as chronic inflammation, metabolic reprogramming and tumorigenesis		
PDIA4 [b]	protein disulfide isomerase family A member 4	Its expression is upregulated by the ATF6-CREB3L3 pathway	↑ / ↓	↓ / =
PGC1a [b]	peroxisome proliferator-activated receptor gamma coactivator 1-alpha	Transcription factor that is the master regulator of mitochondrial biogenesis	= / ↓	= / =
pIpC	polycytidylic acid	Immunostimulant used to simulate viral infections and which activates *Mx1-Cre*$^{+}$		
sod2/sod1 [b]	superoxide dismutase-2/superoxide dismutase-1 ratio	Measure of mitochondrial oxidative stress, being the ratio of the gene expression of the mitochondrial antioxidant enzyme SOD2 and that of the cytoplasmic antioxidant enzyme SOD1, which both catalyse the conversion of the superoxide radical into oxygen and hydrogen peroxide	↑ / =	= / ↑
TFAM [b,c]	mitochondrial transcription factor A	Key activator of mitochondrial biogenesis, involved in mitochondrial	↓ / ↓	= / =

		transcription as well as mitochondrial genome replication		
UPR	unfolded protein response	Cellular stress response related to endoplasmic reticulum stress		
XBP1s [b]	X-box binding protein 1, spliced form	XBP1 is a transcription factor that regulates the expression of cellular stress response genes; its activation requires splicing of its mRNA, resulting in spliced X-box binding protein 1 (XBP1s)	↑ / =	↓ / =

The impact of critical illness or of autophagy deficiency during critical illness on the molecular markers quantified in this study are indicated with "=" when there was no effect", with "↑" for an increase and with "↓" for a decrease. [a] DNA content, [b]: evaluated by mRNA expression, [c]: evaluated by protein expression, d: evaluated by activity.
